# MRI for Crohn's Disease: Present and Future

**DOI:** 10.1155/2015/786802

**Published:** 2015-08-27

**Authors:** Kichul Yoon, Kyu-Tae Chang, Hong J. Lee

**Affiliations:** ^1^Department of Internal Medicine, Korea University, Seoul 136-705, Republic of Korea; ^2^Biomedical Research Institute, Chung-Ang University College of Medicine, Seoul 156-756, Republic of Korea; ^3^National Primate Research Center (NPRC), Korea Research Institute of Bioscience and Biotechnology (KRIBB), Ochang 363-883, Republic of Korea

## Abstract

Crohn's disease (CD) is a chronic inflammatory condition with relapsing-remitting behavior, often causing strictures or penetrating bowel damage. Its lifelong clinical course necessitates frequent assessment of disease activity and complications. Computed tomography (CT) enterography has been used as primary imaging modality; however, the concern for radiation hazard limits its use especially in younger population. Magnetic resonance (MR) imaging has advantages of avoiding radiation exposure, lower incidence of adverse events, ability to obtain dynamic information, and good soft-tissue resolution. MR enterography (MRE) with oral contrast agent has been used as primary MR imaging modality of CD with high sensitivity, specificity, and interobserver agreement. The extent of inflammation as well as transmural ulcers and fibrostenotic diseases can be detected with MRE. Novel MR techniques such as diffusion-weighted MRI (DWI), motility study, PET-MRI, and molecular imaging are currently investigated for further improvement of diagnosis and management of CD. MR spectroscopy is a remarkable molecular imaging tool to analyze metabolic profile of CD with human samples such as plasma, urine, or feces, as well as colonic mucosa itself.

## 1. Introduction

Inflammatory bowel disease (IBD) includes two major forms of chronic intestinal disorder: Crohn's disease (CD) and ulcerative colitis (UC) [1, 2]. CD is a chronic inflammatory condition characterized by relapsing-remitting clinical behavior, potentially affecting any portion of the gastrointestinal tract from mouth to anus. It can occur at any age but most often in second or third decade [3]. A systematic review revealed that the highest annual incidence of CD was 12.7 per 100,000 person-years in Europe, 5.0 person-years in Asia and the Middle East, and 20.2 per 100,000 person-years in North America [4]. According to an extensive review of natural course for CD [5], at the time of diagnosis one-third of the patients had ileitis, colitis, or ileocolitis, up to one-third of the patients had evidence of a stricturing or penetrating intestinal complication. Half of all patients had experienced an intestinal complication within 20 years after diagnosis. The combined effects of genetic, environmental, and/or epithelial barrier dysfunction cause activation of mucosal immune responses, which in turn lead to inflammatory response [1]. It is not unusual to find intestinal inflammation leading to progressive bowel damage, increasing disability, and an impaired quality of life [5].

The diagnosis of CD is made from patient's history and physical examination supported by laboratory, endoscopic, radiologic, and pathologic findings. The European Crohn's and Colitis Organisation (ECCO) grouped clinical disease activity into mild, moderate, and severe but these are not precisely defined entities. Most clinical trials in patients with active Crohn's disease recruit patients with a Crohn's Disease Activity Index (CDAI) of >220 [6]. This index is a point score and comprises eight items (stool frequency, abdominal pain, subjective general wellbeing, presence of complications and abdominal mass, use of antidiarrheal medications, hematocrit, and weight deviation) [7]. Treatment of Crohn's disease aims to achieve sustained clinical and endoscopic remission and to interrupt the disease course that ends in intestinal failure and complications [8].

## 2. MRI for CD: Present

### 2.1. Diagnostic Tools for CD

The gold standard diagnostic tools for CD are ileocolonoscopy and gastroduodenoscopy, providing direct and reliable image of mucosal surface. They have advantage of getting the tissue sample and even treating bleeding complication. However, they only cover proximal small bowel or terminal ileum, requiring other modalities for small bowel evaluation. Transabdominal ultrasound is used as initial imaging modality but it lacks both sensitivity and specificity with high interobserver variability [2]. Capsule endoscopy provides mucosal imaging of small bowel but can only be used when stricture is excluded. Patients with extensive small bowel CD are at higher risk of capsule retention, limiting its clinical use [9, 10]. There have been several radiologic approaches to assess small bowel in CD. The small-bowel follow-through (SBFT) has been the standard modality; however, several studies have shown that SBFT is not accurate and feasible study over other modalities such as computed tomography (CT), enterography, or capsule endoscopy [11, 12]. Among them, CT enterography has been the most commonly used cross-sectional imaging modality to evaluate CD patients. However, due to its potential hazard from ionizing radiation, its repetitive use can be of concern especially in CD, a disease with early onset and frequent relapses [13, 14].

Magnetic resonance (MR) imaging is being preferred because it lacks radiation exposure with validated sensitivity and specificity in both adults and children [15–18]. MR imaging has many advantages other than the lack of radiation, such as provision of static and dynamic three-dimensional information of small bowel, improved soft-tissue contrast resolution, and lower incidence of adverse events compared with CT with iodinated contrast agent. On the contrary, it also has limitations such as higher cost, variations in image quality, and lower spatial and temporal resolution [17, 19–22].

### 2.2. MR Enterography (MRE)

#### 2.2.1. Intraluminal Contrast Agents: Type of Contrast

Use of MRI for bowel has not been popular until recently because it needed long acquisition time, making the imaging of the primarily peristaltic organ difficult. Furthermore, lack of proper contrast for bowel had also limited its use. Imaging of small bowel needs luminal distension because even large lesion can be undetected if the bowel is evaluated in collapsed state [23, 24]. According to these signal properties, agents can be classified as positive (“bright” lumen), negative (“dark” lumen), or biphasic agents (“bright” lumen on T1 and “dark” on T2 or conversely “dark” lumen on T2 and “bright” on T1) [23]. There are a number of hyperosmolar T1 hypointense/T2 hyperintense biphasic oral contrast agents currently available. Water is cheap, safe, and readily available agent. However, its rapid absorption limits its role of proper distension of small bowel [25]. So it is mixed with other agents to improve luminal distension. Lactose delays the absorption of the water in contrast material effectively, obtaining conspicuity of the bowel [26]. Diatrizoic acid (gastrografin) can achieve very good distention, homogeneity, and delineation in the central segments from the ileum to the left colon flexure in majority of cases, due to the adequate contrast media supply in these regions. Diarrhea is a major problem affecting nearly all patients [27]. Recent report showed that 3% sorbitol and a psyllium based bulk fiber showed no significant difference at distending the small bowel [28]. Other groups reported that novel mixture containing methylcellulose powder with water, low-concentration (4.9%) barium, and sorbitol allowed good-quality enterographic images and patient tolerance [29].

#### 2.2.2. Intraluminal Contrast Agents: Methods of Delivery

Enteroclysis has been employed as one of the useful MR techniques for evaluation of CD. However, administration of 1.5 to 2 L of isosmotic water solution through nasojejunal catheter causes patient discomfort. In contrast, MR enterography (MRE) takes the cross-sectional images targeting small bowel after administration of large volume of oral enteric contrast without nasojejunal intubation [20, 30]. Several studies have shown that MRE has better patient compliance than enteroclysis and similar diagnostic efficacy to the method in evaluating CD [31, 32]. It assesses not only the bowel but also the surrounding perienteric structures such as mesentery or adjacent organs [19, 20, 22, 33]. Therefore, proper use of aforementioned contrast agents does allow MR imaging evaluation of small bowel CD.

### 2.3. Conventional Techniques

Antiperistaltic agents are commonly used these days in practice as recent studies have shown benefits in using antiperistaltic (IV glucagon), improving visualization of the bowel wall, mainly because they suppress peristalsis to reduce motion-related blurring and ghosting artifacts [34].

Although there is no definite consensus or guideline on oral contrast regimen, the agents described above are generally administered 10 to 15 minutes before the scanning. The scout imaging assesses the progression of oral contrast and distension. If the contrast has not reached terminal ileum, the further imaging can be postponed. Patients can be scanned in the prone or supine position in multichannel torso or body phased array coil on a 1.5- or 3-tesla (T) magnet [18]. The sequences of the MRE examination include axial and coronal T2-weighted single-shot fast spin echo (SSFSE) sequences and balanced steady state free precession (SSFP) sequences. SSFSE acquires all the necessary data for reconstruction with one excitation [35]. It is excellent for visualizing edema, wall thickening, and fluid in bowel wall and mesentery [18]. Balanced SSFP is characterized by two unique features, a very high signal-to-noise ratio and a T2/T1-weighted image contrast [36]. The recent development of faster pulse sequences provides an opportunity to provide a movie of cine images [37]. Cine imaging confers the ability to observe the motion of intestinal segments over a relatively short period and in real time. It provides high temporal, spatial, and contrast resolution for monitoring bowel contractions [38]. After administration of IV gadolinium-containing contrast material (0.1-0.2 mmol/kg), dynamic coronal 3D T1-weighted gradient echo (GRE) sequences with fat suppression are obtained in time intervals of 45 to 55, 70, and 180 seconds. These intervals are institutionally specific. Delayed axial and coronal postcontrast 2D or 3D T1-weighted sequences with fat suppression are acquired following dynamic imaging [18]. Although rapid transit to the right colon is seen in some patients, most patients require a delay of at least 40–60 minutes from contrast material ingestion to imaging [39, 40].

The field strength of the MRI magnet will affect the rate at which images may be obtained [37]. For 1.5 T systems, the MRI technologist selects 5 to 7 representative coronal slices of the abdomen using the sagittal localizer image. Each slice is obtained during a 30-second period with a total of 50 images obtained during that time period. With newer 3 T systems, it is possible to obtain up to 110 images per location during the same 30-second time period. The presence of “banding” artifact inherent to the SSFP sequence is of concern for 3 T imaging. It is pronounced particularly at air/soft tissue interfaces [37]. Imaging at higher field strength has a greater signal-to-noise ratio and also has the potential of reducing scan times. A retrospective study of 46 children with biopsy-proven CD reported that, with appropriate attention to technique and with optimal distension and control of movement, high-quality 3 T assessment of the abdomen, pelvis, and perineum is possible [41].

### 2.4. Diffusion-Weighted MRI (DWI)

Like other abdominal applications, DWI benefits from increased signal-to-noise ratio at 3 T with improved sensitivity compared with 1.5 T. However, image distortion from increased magnetic susceptibility often results in a loss of image quality at 3 T. Magnetic susceptibility artifacts may be limited by using parallel imaging techniques such as sensitivity encoding, integrated parallel acquisition, generalized autocalibrating partially parallel acquisition, and array spatial sensitivity encoding [42].

Diffusion-weighted MRI (DWI) has long been used in other parts of body such as brain. Although application of DWI to assess bowel is a relatively new trend, DWI may yield comparable performances for detecting and assessing ileal inflammation in CD [43]. A high signal intensity in DWI and restricted diffusion of the bowel wall also have been related to acute inflammation [44–46].

A review with 18 patients having active CD of terminal ileum showed that DWI can provide quantitative measures of small bowel inflammation that can differentiate actively inflamed small bowel segments from normal small bowel in CD. It showed better sensitivity compared with dynamic contrast-enhanced MR [46]. An observational prospective study with 130 CD patients reported that, at certain apparent diffusion coefficient, sensitivity and specificity of discriminating active from nonactive CD were 96.9% and 98.1%, respectively, for the colon/rectum, and 85.9% and 81.6%, respectively, for ileum. They also reported high interobserver agreement [47]. A recent study involved 31 CD patients with ileal involvement to compare DWI with conventional MRE in estimating inflammation in small bowel CD. DWI hyperintensity was highly correlated with disease activity evaluated using conventional MRE [43]. DWI also showed additional value to T2-weighted imaging for diagnosis of internal fistula and sinus tracts, according to a retrospective study reviewing the 25 fistulous lesions [48].

### 2.5. MRE Findings in CD

Patients with CD can be classified by Montreal or Paris classification regarding age of onset, localization, behavior, and growth. The behavior is subdivided into B1 (nonstricturing/nonpenetrating), B2 (stricturing), and B3 (penetrating). Perianal penetrating diseases are considered separately, as they show different prognosis than other penetrating patterns of CD [49]. Although there is no exact definition or consensus, disease activity is usually grouped into mild, moderate, and severe. CDAI comprises relatively complex clinical and laboratory data, limiting its clinical use [2]. Differentiation between the subtypes is clinically important because active inflammation is usually treated medically unless there are extramural complications, while fibrostenotic disease characterised by obstructive symptoms often requires surgery [50].

Maglinte et al. suggested an imaging-based classification of small bowel CD subtypes. They radiologically classified CD into four groups: active inflammatory, fibrostenotic, fistulizing/perforating, and reparative or regenerative subtype. They reasonably correlate with the clinical classifications [22].

### 2.6. Active Inflammatory Subtype

Active inflammatory subtype of CD is characterized by local inflammation, aphthoid and deep ulcers, frequent transmural inflammation with lymphoid aggregates, and granuloma formation. Different morphological and functional parameters are used to assess disease activity in MRE. They are thickness of wall, the degree of wall gadolinium- (Gd-) enhancement, T2 mural signal intensity, enhancement of local lymph nodes, pattern of wall Gd-enhancement, increased mesenteric vascularity, and time-enhancement curves of Gd-wall enhancement. Each of these parameters has proved to be statistically correlated with the biological, endoscopic, or histological activity [21, 51–53].

Mesenteric edema is present in some patients with advanced active disease, and it tracks along the adjacent mesentery from an inflamed bowel loop [40]. The degree of thickening has been proven to be correlated with Crohn's Disease Activity Index. A wall thickness greater than 3 mm in a distended small bowel loop can be regarded as abnormal. In patients with small bowel CD, wall thickness usually ranges between 5 and 10 mm. Thickened wall without edema has low to moderate signal intensity on SSFP and HASTE images [40, 54]. Stratified contrast enhancement with avid enhancement of the mucosa relative to the submucosa and muscular layers helps confirm active Crohn's disease [40]. Signal hyperintensity in the bowel wall in T2-weighted images (T2WIs), especially in fat-suppressed sequences, indicates wall edema and is a sign of acute inflammation [55]. Increased intravenous contrast enhancement of the bowel wall also indicates acute inflammation. Mucosal increased enhancement with submucosal edema is so-called “stratified type of bowel enhancement” and has been especially related to acute disease [30]. Full-thickness nonstratified enhancement of intestinal wall can represent transmural acute inflammation as well [56].

On high-resolution SSFP image with fat suppression, aphthous ulcers are seen as a nidus of high signal intensity surrounded by moderate signal intensity [57]. Transmural ulcers are outlined by luminal contrast material and seen as linear high-signal intensity into the bowel wall. Images obtained in a plane perpendicular to the bowel allow accurate assessment of transmural and peri-intestinal inflammatory changes [57].

### 2.7. Fibrostenotic/Fistulizing Subtype

Small bowel obstruction is the chief clinical manifestation of fibrostenotic disease. A fixed narrowing of the affected segment is seen on MRE. Chronic fibrotic strictures typically are hypointense on both T1- and T2-weighted images and show inhomogeneous contrast enhancement, with no evidence of edema or surrounding inflammation of mesentery [20, 57]. Large sinus tracts and fistulas may be outlined by enteral contrast material and are seen as high-signal intensity linear features. Solitary internal fistulas present as tubular tracts, star-shaped bowel loops, indicating a complex internal fistula [58].

A recent study with MRE evaluating 76 CD patients showed high *κ* value and Lin's concordance correlation coefficient between the intraoperative and radiological assessments. The diagnosis of a stricture had highest sensitivity and the detection of inflammatory mass showed the lowest sensitivity. Abscesses had the lowest positive predictive value in that study, while fistulae were found to have the best correlation between the surgical and MRE-based diagnoses [59]. Various efforts have been made to improve diagnostic value of MRE. A study comparing MRE with or without water enema showed that MRE with enema was statistically superior to MRE without enema in detecting inflammation in the terminal ileum, ascending colon, and rectum [60]. Further improvement of imaging quality, sensitivity, and specificity of MRE is expected with technical developments.

### 2.8. MRI for Motility

Many studies have shown that gut motility at MRI is decreased in active or chronic CD. The bowel segments affected by CD show significantly increased number of lesions in individual patient as well as overall patients with CD [61–64]. The sequence for small bowel motility is a fast cine sequence using fast T2-weighted SSFP or echo planar imaging sequences with a maximum repetition time of 1 second. The images must be acquired before the application of a spasmolytic drug [61]. There was a study correlating MR-detectable motility alterations of the terminal ileum with biopsy-documented active and chronic changes in CD. It analyzed 43 patients who underwent both MRE and terminal ileum biopsy. Histopathology correlated with presence of hypomotility or complete arrest and grade of motility alterations [63]. Another study measured contraction frequency, amplitude, amplitude diameter ratio, and luminal diameter via MRI as well as the blood levels of CRP and fecal levels of calprotectin. A significant inverse linear correlation was found between the contraction frequency and both the level of CRP and calprotectin [64]. In addition, a study with healthy volunteers assessing software-quantified small bowel motility captured with MRI and testing the ability to detect changes in motility induced by pharmacologic agents showed that the repeatability between baseline measurements of motility was high. The measured motility with neostigmine was significantly higher than that with placebo, whereas that with butylscopolamine was significantly lower than that with placebo [65].

## 3. MRI for CD: Future

### 3.1. PET-MRI

There are novel MRI-related techniques not yet easily available or cost-effective but have potential application to CD. Both positron emission tomography (PET) with fluorodeoxyglucose (FDG) and MRI have been shown to be useful for diagnostic evaluation of a variety of inflammatory processes. However, only a few PET-MRI units are operational around the world and mostly only for research use. CD could be a candidate target of the novel technique PET-MRI but it currently has no clearly established role [66].

### 3.2. MR Spectroscopy

#### 3.2.1. Introduction of MRS

Genomics and proteomics have emerged to explain biological phenomenon. However, they do not provide dynamic metabolic status of tissue and whole organism [67]. MR molecular imaging and MR spectroscopy (MRS) are still experimental but promising, because they are some of the leading technologies in metabolomics and have possibility to analyze and characterize the molecular composition of inflamed bowel wall [51]. As MRS has more accurate quantitation and better reproducibility than mass spectrometry, MRS is already routinely used in many malignant conditions such as brain, breast, and prostate cancer [68]. The essential goal of MRS is to determine the distribution of metabolites associated with the relevant pathology. Their presence, absence, or relative amount compared with other metabolites is analyzed [69]. The MR signal produces varying but predictive pattern of resonant frequencies corresponding to molecular arrangements of some atomic nuclei susceptible to perturbations, typically protons. The structural or chemical information regarding the reaction of the nuclei can be obtained. After the examination is performed the data are usually presented in a one-dimensional NMR (nuclear MR) frequency spectrum [69, 70]. There are a number of biologically relevant MR-visible isotopes in vivo. The most common nuclei used are those that do not require exogenous label such as ^31^P, ^1^H, and ^23^Na which generate spectra from endogenous metabolites [71].

#### 3.2.2. MRS Techniques in CD

^1^H NMR is most commonly employed in MRS for inflammatory bowel disease. On a one-dimensional NMR spectrum, the peak shows signal from a particular chemical configuration of the nucleus (e.g., ^1^H) and the intensity is also noted. Area under a peak relates to the number of nuclei that have identical chemical bonding configuration [69]. Recently, two-dimensional J-resolved (JRES) NMR spectroscopy has been introduced. It additionally disperses the overlapping resonances into a second dimension. It has advantages such as increased spectral dispersion, confidence in metabolite identification, and reduced batch-to-batch variation. It also has some disadvantages like longer acquisition times, higher technical variability, and phase-twisted lineshapes [72].

Chemometric analysis and comparison of ^1^H NMR are commonly hampered by intersample peak position and line width variation due to matrix effects. To mitigate this problem, “targeted profiling” method was introduced. Individual NMR resonances of interest are mathematically modeled from pure compound spectra. This database is then interrogated to identify and quantify metabolites in complex spectra of mixtures, such as biofluids. The method is highly stable in PCA-based pattern recognition, insensitive to water suppression, relaxation times, and scaling factors. Hence, direct comparison of data acquired under varying conditions is made possible [73].

The feasibility of metabonomics in clinical studies was first suggested using ^1^H NMR based metabonomic analysis on plasma and urine samples obtained from healthy subjects. The ^1^H NMR spectra obtained for urine and plasma samples were analysed using principal components analysis (PCA) in order to generate metabonomic data [74, 75]. ^1^H NMR-based metabonomic approach has been suggested as a quantitative measurement of metabolic response in CD [76].

A recent study tried to find metabolic biomarkers and the correlation between serum zinc in CD patients performed ^1^H NMR spectroscopy experiments on a 500 MHz spectrometer and five-millimeter NMR tubes. Deuterium oxide (D_2_O) 100 *μ*L provided NMR lock signal for NMR spectrometer. Broad resonances caused from combination of high molecular weight components were suppressed by Carr-Purcell-Meiboom-Gill (CPMG) experiment. It enhanced visualization of superimposed sharper resonances from low molecular weight (amino acids and carboxylic acids). CPMG spin echo pulse sequent was used to record 1D ^1^H NMR spectra, which were recorded at 298 K. Peaks in the serum spectra were referenced to the chemical shift of lactate. The integral values of each spectrum were normalized to a constant sum of all integrals [77].

Biochemical analysis of fecal extracts has been studied by several institutions because it is cost-effective and reflects biochemical changes of bowel disease. Characterization of fecal extracts obtained from patients with CD and UC by employing ^1^H NMR spectroscopy and multivariate pattern recognition techniques was reported to differentiate two IBDs. The 400 *μ*L of fecal extract was added to 200 *μ*L of water containing D_2_O and a chemical shift reference sodium 3-(trimethylsilyl)propionate-2,2,3,3-d_4_. After centrifuge, the supernatant was pipetted into 5 mm NMR tube and ^1^H NMR spectra were acquired for each sample at 600.13 MHz for ^1^H equipped with a 5 mm triple resonance probe with an inverse detection [78].

### 3.3. MRS Findings in IBD

There have been several studies on MRS findings in IBD. Most of them included both UC and CD for analysis, showing similarities and differences between them. A study using in vitro ^1^H NMR reported that patients with IBD showed similar metabolic profile in macroscopically involved and uninvolved colonic mucosa compared with that of controls [76]. The past few years have seen an increase in studies of experimental and human IBD focusing on the search for small metabolites, such as amino acids, bases, and tricarboxylic acid (TCA) cycle intermediates. Experimental methods for the screening of metabolites in serum, urine, fecal extracts, and colon tissue include ^1^H NMR spectroscopy [79]. A very recent study tried to search for metabolic biomarkers and the correlation with serum zinc in Crohn's disease patients. The result suggested valine and isoleucine as differentiating metabolites for CD diagnosis [77]. The authors have previously proposed that ^1^H NMR could be used as part of metabonomics to diagnose CD, as the disease shows signs and symptoms similar to other medical problems. Applications of NMR and supervised pattern recognition in the field of metabonomics were also reviewed in recent years [80].

According to an analysis of the fecal extracts of both CD and UC patients, they were characterized by reduced levels of butyrate, acetate, methylamine, and trimethylamine, reflecting changes in the gut microbiota. Quantities of amino acids were elevated in the feces from CD and UC, implying malabsorption. Metabolic differences in fecal profiles were more marked in the CD group, indicating more extensive inflammation in the specific study. They reported that glycerol resonances were a dominant feature of fecal spectra from patients with CD [78]. A basic research also supports the metabonomic approach to IBD. Metabolic profiling of the fecal extracts of dextran sulfate sodium- (DSS-) induced colitis mice with ^1^H NMR was reported. It was carried out to assess the effects of probiotics on colonic inflammation. Mice treated with probiotic lactic acid bacteria showed increased short chain fatty acids levels in the feces [81].

Quantitative metabonomic profiling of serum, plasma, and urine from human subjects with active CD and UC was also performed, employing ^1^H NMR and “targeted analysis.” In serum and plasma of IBD patients, methanol, mannose, formate, 3-methyl-2-oxovalerate, and amino acids such as isoleucine were prominently increased. In urine, maximal increases were observed for mannitol, allantoin, xylose, and carnitine. Both serum and plasma of UC and CD patients showed significant decreases in urea and citrate, whereas, in urine, decreases were observed, among others, for betaine and hippurate. The metabolic differences between the CD and UC cohorts are less pronounced [82].

To identify tissue-specific markers associated with CD, a metabonomic approach to monitor events associated with the gradual development of CD-like ileitis in the TNF(ΔARE/WT) mouse model was done using ^1^H NMR. The approach showed shifts in the intestinal lipid metabolism concomitant to the histological onset of inflammation. The advanced disease was characterized by a significantly altered metabolism of cholesterol, triglycerides, phospholipids, plasmalogens, and sphingomyelins in the inflamed ileal tissue and the adjacent proximal colon. Modifications of the general cell membrane composition, alteration of energy homeostasis, and the generation of inflammatory lipid mediators could explain the result [83].

Metabolism of the colonic mucosa itself of patients with IBD was also reported using ^1^H NMR. In the active phase of UC and CD, significantly lower concentration of amino acids (isoleucine, leucine, valine, alanine, glutamate, and glutamine), membrane components (choline, glycerophosphorylcholine, and myoinositol), lactate, and succinate was observed compared to normal mucosa of controls. Patients in the active phase of UC and CD also showed increased level of alpha-glucose compared to normal mucosa. In contrast to active disease, altered level of metabolites indicated decreased protein and carbohydrate metabolism in patients with chronic inflammation. Decreased energy status and deterioration of mucosa integrity during chronic inflammation could explain these findings [84]. In a study based on urinary metabolomics, individuals with IBD could be differentiated from healthy ones. Major differences between IBD and healthy included TCA cycle intermediates, amino acids, and gut microflora metabolites [85].

NMR has shown possibilities to differentiate between UC and CD, which is not always easy on clinical practice. Interestingly, formate was significantly lower in colonic mucosa of patients with active UC compared to patients with the active colonic CD, suggesting the potential of in vitro MRS in the differentiation of these two diseases [84]. NMR of urine samples also revealed that hippurate levels were lowest in CD patients and differed significantly between the three cohorts (UC, CD, and healthy control). Urine formate levels were higher and 4-cresol sulfate levels were lower in CD patients than in UC patients or controls. PCA also revealed clustering of the groups; PLS-DA modeling was able to distinguish the cohorts [86].

### 3.4. Nanoparticles

Imaging of inflammatory sites can be achieved by making use of several different characteristics of affected tissues. A relatively new and promising application of lipidic nanoparticles is their use as multimodal MR contrast agents. The imaging of inflammatory sites has been studied mainly in cardiovascular diseases such as atherosclerosis or myocardiac infarction. Nanoparticles are employed not only for diagnosis but also for monitoring of drug delivery [87, 88]. As many inflammatory conditions have distinct molecular features in the diseased tissues, lipid-based nanoparticles could be another possibility to evaluate CD.

Imaging of inflammatory sites can be achieved by making use of several different characteristics of affected tissues. Specific overexpression of endothelial adhesion molecules caused by the inflammatory cytokines can be used as a target for contrast agents. Ongoing angiogenic response could also be used by injection of a nonspecific contrast agent which would accumulate at the inflamed site. Magnetic nanoparticles have frequently been used as MRI contrast agents as they disturb the relaxation of nearby protons, thus darkening T2-weighted MRIs. Depending on their size, nanoparticles can be used to detect vascular leak. There was a report on development of a noninvasive method using ferumoxtran-10 nanoparticles to visualize type 1 diabetes at the target organ level in patients with active insulitis. Fermoxtran-10 has a dextran coating and it is readily taken up by macroophages without provoking activation or inducing proinflammatory cytokines. It has been used in the noninvasive detection of clinically occult cancer metastatic to lymph nodes. All participants underwent at least 3 MRI scans: a premagnetic nanoparticle (MNP) series, an immediate post-MNP series, an indicator of vascular volume and useful for pancreas volume estimates, and a delayed post-MNP series, which likely reflects leakage of MNPs and retention by phagocytic cells [89, 90].

In addition, migration of cells involved in inflammation can be followed after labeling the cells with an appropriate contrast material when cells are labeled outside the body and subsequently injected [91, 92]. Currently, much effort is being put into research on the targeted imaging of cell adhesion molecules involved in inflammation. Targeting of the adhesion molecule could be done with antibodies, proteins, peptides, or small molecules conjugated to an MRI contrast agent. Lipid-based contrast agents have been used for those strategies. However, there have not been enough studies to apply this strategy specific to CD [93, 94].

Using fluorescent magnetic nanoparticles, a group of researchers screened the library against different cell lines and discovered a series of nanoparticles with high specificity for endothelial cells, activated human macrophages, or pancreatic cancer cells [95]. Currently the studies using nanoparticles particularly for CD have been scarce. It could be a topic for researchers; however it currently has no clearly established role in CD.

## 4. Conclusion

In recent years, MRE has become a part of standard diagnostic modality in CD. Novel MRI techniques such as DWI, motility studies, PET-MRI, and molecular imaging might further contribute to diagnosis and management of this chronic inflammatory disease.
